# From the “Dark Days” to Golden Days: Lessons From the Botswana National Validation Committee's Journey on the Path to Elimination of Vertical Transmission of HIV

**DOI:** 10.1002/jia2.70105

**Published:** 2026-06-19

**Authors:** Mogomotsi Matshaba, Queen Nthusang, Jessica Setswalo, Tebogo Madidimalo, Bornapate Nkomo, Mooketsi Molefi

**Affiliations:** ^1^ National Validation Committee, Ministry of Health Gaborone Botswana; ^2^ Botswana‐Baylor Children's Clinical Centre of Excellence Gaborone Botswana; ^3^ Ministry of Health Gaborone Botswana; ^4^ World Health Organization, Botswana Country Office Gaborone Botswana; ^5^ Faculty of Medicine, Department of Family Medicine & Public Health University of Botswana Gaborone Botswana

1

The global HIV epidemic is now mature, potentially getting closer to elimination targets set by UNAIDS and the path set by the World Health Organization (WHO) [1]. In parallel, WHO has advanced the concept of triple elimination of mother‐to‐child‐transmission (MTCT) of HIV, syphilis and hepatitis B, recognizing the shared service delivery platforms, health system enablers and human rights principles to eliminate these infections among pregnant women and infants. Lessons from Botswana demonstrate that elimination is achievable when key ingredients are in place, including adequate resource allocation, political will, community and stakeholder engagement, and evidence‐based programming, among others [2].

The first country to be validated by WHO for eliminating MTCT of HIV was Cuba in 2015, followed by Thailand, Armenia and Moldova in 2016 [3]. These early validations occurred in countries with low HIV burden. In contrast, countries with a high burden of HIV—and overlapping burdens of syphilis and hepatitis B—face substantially greater barriers to achieving elimination. These challenges include large populations of people living with HIV, persistent structural and developmental constraints, competing national priorities and limitations in health systems capacity, financing, and programme implementation [4].

Given these challenges facing countries with a high burden of HIV, the WHO developed a path to elimination (PTE) of transmission of HIV, making it possible for these countries to celebrate each step of the process [1]. The process is overseen by local, regional and global committees that work together to ensure progress. The achievement of elimination of MTCT of HIV requires the Ministry of Health (MoH) ownership, rigorous data analysis and intensive programme assessment. This involves a set of impact and process indicators looking at data quality, programme strength, laboratory quality and human rights, gender equality and community engagement. see Figure 1.

**FIGURE 1:**
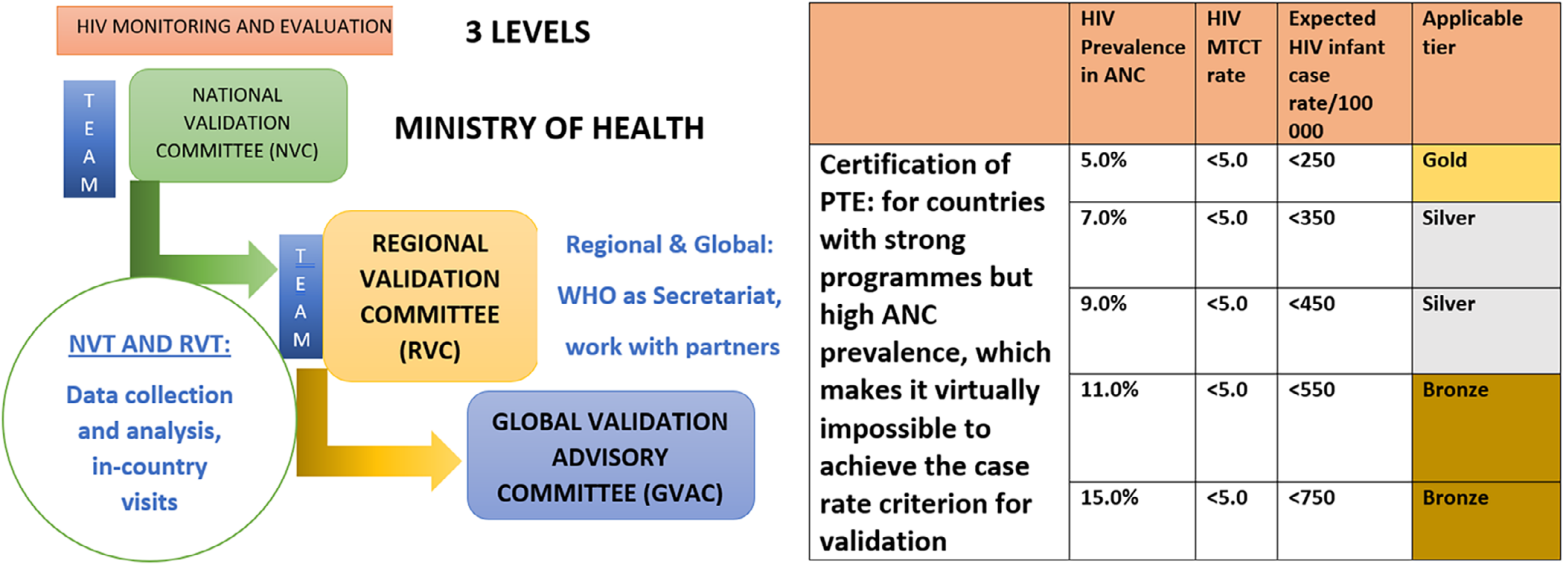
(Left) Summary of the process of path to elimination validation, as described by the WHO. (Right) Scenarios of targets to be met over the prescribed period by applicants [5]. Abbreviations: ANC‐Antinatal Clinic; MTCT‐mother to child transmission NVT‐National Validation Team; PTE‐ Path to Elimination; RVT‐Regional Validation Team.

Botswana has made remarkable progress since the period in which Former President Festus Mogae warned at the 2001 UN General Assembly that the nation faced the threat of extinction [6]. This was followed by significant government investments in the nation's health and an outpouring of solidarity and support from the international community, and subsequently led to Botswana being one of the first high HIV‐prevalence countries to achieve the WHO silver‐tier validation for the PTE of HIV in 2021 [7]. Sustaining this progress and advancing to gold‐tier status in 2025 required strengthening health systems, optimizing service delivery and addressing residual programmatic gaps.

WHO first established National Validation Committees (NVC) to assess each country's progress towards elimination targets. In response, the MoH HIV Department, with support from partners, constituted Botswana's NVC in 2017. Serving as the secretariat, the MoH led and coordinated the country's validation process and formally engaged with the WHO Regional Validation Committee for assessing Botswana's certification progress. The committee was comprised of members with different skillsets in paediatrics, infectious diseases, biostatistics and epidemiology, laboratory science, and socio‐legal expertise, and community advocacy, with representation from affected communities. WHO regional and global offices provided support and linked the NVC with counterparts in countries that had undergone the process. These countries provided invaluable input.

The process of validation is labour‐ and resource‐intensive, needing dedication and perseverance. It involves retrospective analysis of programmatic, surveillance and laboratory data for the prescribed time periods. Key indicators assessed include antenatal care (ANC) coverage, maternal HIV testing and antiretroviral therapy initiation rates, vertical transmission rates and paediatric HIV incidence. This process followed the nation's initial silver tier validation in 2021 [7]. Notably, Botswana also reached the 95‐95‐95 UNAIDS 2030 targets in the same period [8].

The official report to WHO showed ANC coverage remained above 95%, with HIV testing exceeding 99% and ART coverage for HIV‐positive pregnant women over 98%. Vertical HIV transmission rates decreased from 1.3% in 2020 to 1.2% in 2023. New paediatric HIV acquisitions declined by 27%, while the case rate fell below 250 per 100,000 live births. These are the results that led to the gold‐tier certification of PTE of HIV that was officially awarded by WHO [7].

The validation process was not without challenges that posed major obstacles to the validation process. As a high HIV burden country, the persistently elevated HIV burden among women of childbearing age remains a key barrier to elimination [9, 10]. Sustained viral suppression among all women living with HIV throughout pregnancy and breastfeeding is essential for success. However, suboptimal outcomes among adolescents and young women living with HIV and other key populations continue to undermine progress. Without deliberate and sustained focus on these groups, achieving full elimination will remain unattainable.

Sustained resource mobilization is important, especially now as the global health financing priorities of high‐income nations change. As a middle‐income country, Botswana faces challenges with significant reductions in international donor support, placing a greater burden on domestic resources. Lastly, the rising incidence of non‐communicable diseases, including in the group of ageing people living with HIV, presents a challenge in managing the dual epidemics.

Botswana used the validation process to strengthen systems and incrementally improve service delivery. Summary of lessons learned include the following:

- Government ownership and leadership were critical: Positioning the MoH as the secretariat of the NVC led to early ownership and sustained government buy‐in throughout the validation process
- High‐quality data systems were essential: Accurate data capture and its effective use were fundamental to demonstrating programme performance and meeting validation requirements.
- Integrated programme strengthening was necessary: The “triple elimination” strategy required that HIV, syphilis and hepatitis B programmes be strengthened concurrently, rather than in isolation.
- Community engagement enhanced validation success: Meaningful engagement of civil society and women living with HIV improved the relevance, credibility and acceptability of the validation process.

As next steps, Botswana aspires to achieve full WHO triple elimination status. The nation's progress to gold‐tier PTE of HIV validation demonstrated that this is possible. There needs to be a focus on risk‐based prevention and on rapidly adopting innovations like long‐acting injectables for high‐risk pregnant and breastfeeding women. Closing the paediatric care gap, increasing domestic financing and leveraging public‐private partnerships while investing in research will be essential to sustaining gains and achieving validation for triple elimination of MTCT.

## Author Contributions

The concept for this commentary was developed by MM, QN, JS, TM, BN and MoM. MM wrote the first draft. All authors contributed and approved the final version.

## Conflicts of Interest

The authors declare that they have no conflicts of interest.

## Data Availability

The data that support the findings of this study are available on request from the corresponding author. The data are not publicly available due to privacy or ethical restrictions.
